# Spontaneous repositioning of posterior chamber intraocular lens: a mere coincidence?

**DOI:** 10.5935/0004-2749.20200054

**Published:** 2020

**Authors:** Chong Wern-Yih, Chan Jan-Bond, Menon Sudha, Abu Norlelawati, Ismail Shatriah

**Affiliations:** 1 Department of Ophthalmology, Hospital Tuanku Ja’afar, Negeri Sembilan, Malaysia; 2 Department of Ophthalmology, School of Medical Sciences, Universiti Sains Malaysia, Kelantan, Malaysia

**Keywords:** Mydriasis, Miosis, Lenses, intraocular, Lens subluxation/chemically induced, Lens subluxation/surgery, Postoperative complications, Midríase, Miose, Lentes intraoculares, Subluxa ção do cristalino/induzido quimicamente, Subluxação do cristalino/cirurgia, Complicações pós-operatórias

## Abstract

Despite the recent developments in modern cataract surgery and the application of a vast array of new devices and machines, late in-the-bag intraocular lens dislocation remains a devastating, albeit rare, complication. Various nonsurgical and surgical techniques have been used to manage this complication. We report a case of spontaneous repositioning in the left eye of an anteriorly subluxated in-the-bag intraocular lens. The spontaneous repositioning may have been caused by antagonistic effects related to the topical administration of brimonidine and prednisolone. The dislocation was treated without aggressive manipulation or surgical intervention.

## Abstract (pt)

Apesar dos recentes avanços na cirurgia moderna de catarata e da aplicação de uma ampla gama de novos dispositivos, o deslocamento tardio de uma lente intraocular dentro do saco capsular continua a ser uma complicação devastadora, ainda que rara. Várias técnicas cirúrgicas e não cirúrgicas têm sido usadas para tratar esta complicação. Este é o relato de um caso de reposicionamento espontâneo de uma lente intraocular sub-luxada anteriormente dentro do saco capsular do olho esquerdo. Este reposicionamento pode ter sido causado pelos efeitos opostos da aplicação tópica simultânea de brimonidina e prednisolona. O deslocamento foi tratado sem manipulação agressiva ou intervenção cirúrgica.

## INTRODUCTION

New techniques and devices have drastically improved the safety of cataract operations involving intraocular lens (IOL) implantation. Late in-the-bag dislocation of an IOL is a rare, but potentially severe, complication that can occur after cataract surgery. Surgical intervention for this condition is often challenging, and new complications may arise during surgery.

We report a case of spontaneous repositioning in the left eye of an anteriorly subluxated in-the-bag IOL, secondary to a potential pharmacological effect from topical eye drops.

## CASE PRESENTATION

A 70-year-old man presented with a 3day sudden onset of painless vision-blurring in his left eye. He denied diplopia, floaters, flashes, or visual field defects. There was no history of trauma, and he had no complaints in his right eye. The patient had undergone an uncomplicated phacoemulsification of both eyes 12 years prior to this incident.

The best-corrected visual acuity of the patient was 6/9 in the right eye (refraction of -1.50 DS/-1.25 DC × 80°) and 6/9 in the left eye (refraction of -4.50 DS/ -1.75 DC × 30°). In the right eye, the posterior chamber intraocular lens (PCIOL) was subluxated superiorly behind the iris with vitreous in the anterior chamber (AC). There was no detection of pseudoexfoliation (PEX) material. There was no corneal contact with the PCIOL or the vitreous. The intraocular pressure (IOP) was 32 mmHg. Gonioscopy revealed a 360° Shaffer grade 4 without anomalies. A fundus examination revealed a pale disk with a cup-to-disk ratio of 0.8. The axial length was 24.53 mm. The other findings were insignificant.

**Figure 1:**
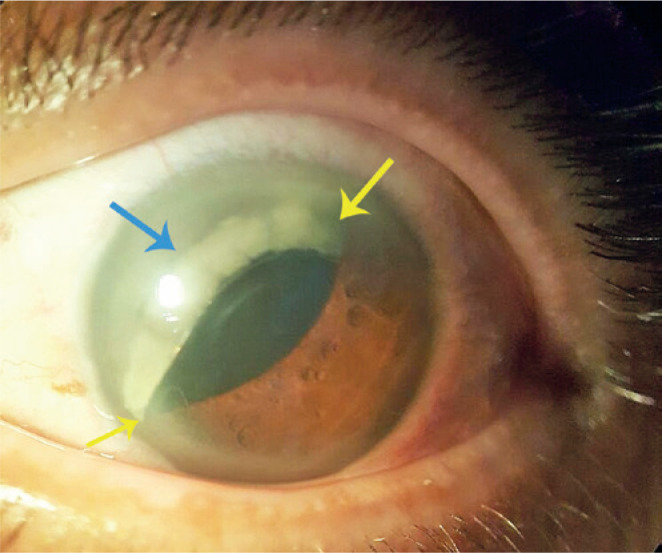
Left eye in-the-bag intraocular lens (blue arrow) was dislocated anteriorly with optic captured by the pupillary margin (yellow arrow).

Examination of the left eye revealed an anteriorly, superiorly subluxated PCIOL that was partially tilted with a prolapsed superior haptic; the optic was captured by the pupillary margin (Figure 1). There was no detection of PEX material. There was no contact between the PCIOL and the cornea, and there was no vitreous in the AC. The AC contained occasional cells. The IOP was 22 mmHg. Gonioscopy revealed a 360° Shaffer grade 4 without anomalies. A fundus examination revealed a pink tilted disc with a cup-to-disc ratio of 0.5. The axial length was 26.29 mm. The other findings were unremarkable.

The preliminary diagnosis for the right eye was primary open-angle glaucoma, superiorly subluxated in-the-bag IOL with vitreous in the AC, and left eye anteriorly, superiorly subluxated in-the-bag IOL with optic-iris capture. A trial treatment of left-eye intensive pupil dilatation with supine positioning for 6h was performed but failed.

Given the raised IOP of the patient, gutta dorzolamide hydrochloride (2%) was prescribed once every 12h for both eyes; gutta brimonidine tartrate (0.2%) was prescribed once every 12 h for the right eye. In view of the mild inflammation, gutta prednisolone acetate (1%) was prescribed once every 6 h for the left eye. Surgical repositioning of the subluxated in-the-bag IOL was planned for the left eye. However, 3 weeks later, the patient reported spontaneous resolution of the vision-blurring in the left eye after waking from sleep.

**Figure 2:**
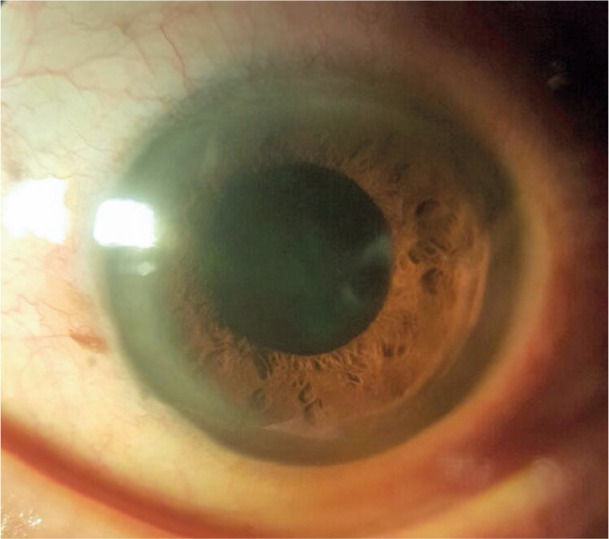
Left eye spontaneous repositioning of intraocular lens.

**Figure 3:**
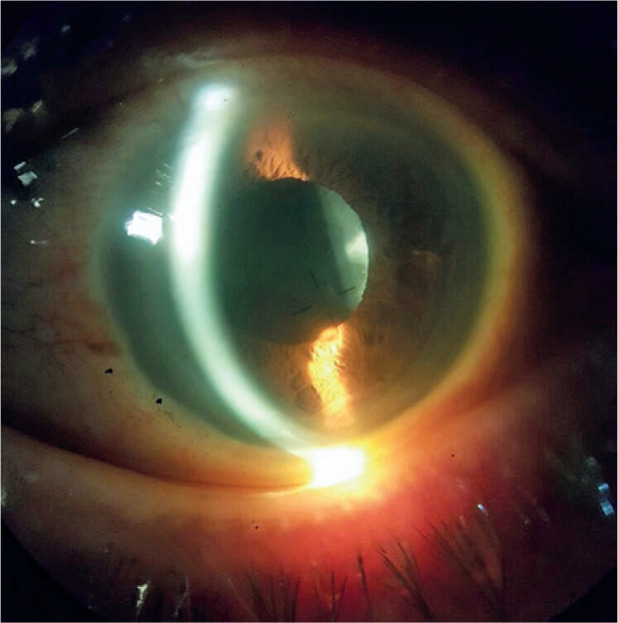
Left eye spontaneous repositioning of intraocular lens.

Examination revealed that the in-the-bag IOL was spontaneously repositioned to the posterior chamber. The pupil in the left eye was round and reactive with a deep and quiet AC (Figures 2 and 3). The IOP was 31 mmHg. Mild pseudophacodonesis was noted. The visual acuity of the patient, assessed using the pinhole test, was 6/9. He received gutta dorzolamide hydrochloride (2%) once every 12h, gutta brimonidine tartrate (0.2%) once every 12h, and gutta latanoprost (0.005%) once at night for both eyes. Following 1 month, an examination revealed a stable PCIOL with an IOP of 10 mmHg.

## DISCUSSION

In-the-bag IOL dislocation is caused by progressive zonular weakening and dehiscence that can occur years after an uncomplicated cataract operation^(1)^. The risk factors for IOL dislocation include PEX, high myopia, connective tissue disorders, glaucoma, previous vitreoretinal surgery, uveitis, and a previous history of trauma^(2)^.

Options for the management of anterior dislocation of an in-the-bag IOL include observation, intensive mydriasis (with or without external manipulation) followed by pharmacological miosis, use of a laser to create a mechanical shock wave for IOL retropulsion, and, if all other strategies fail, surgical repositioning of the IOL^(3)^. Various surgical techniques have been described in the literature, including scleral fixation, iris suture, exchange of the IOL for an AC IOL, iris-claw IOL, and glue IOL^(1,2,4)^.

Prednisolone acetate is a glucocorticoid with up to five-fold anti-inflammatory potency of hydrocortisone. There were reports that, when administered preoperatively, eye drops containing glucocorticoids (prednisolone acetate and dexamethasone acetate) maintained mydriasis^(5-7)^. However, these studies found that administration of glucocorticoids alone did not initiate mydriasis.

Brimonidine is an alpha-2 adrenergic agonist that is used for the treatment of glaucoma. Previous studies have demonstrated that brimonidine tartrate (0.15%) exerts a significant miotic effect under all three illuminance conditions (i.e., scotopic, mesopic, and photopic)^(8)^. While brimonidine tartrate effectively decreased the diameter of the scotopic pupil, it did not significantly change the size of the photopic pupil. This effect that may benefit post-refractive surgery patients who complain of halos and glaring associated with dilated pupils^(9)^. It was reported that a contralateral pupil may react to brimonidine administered on the opposite eye, causing miosis^(10)^.

In the present case, we postulated that the alternation between the mydriasis in the scotopic condition (sustained by the administration of prednisolone) and the brimonidine-induced miosis caused a drastic fluctua tion in the size of the pupil. This fluctuation, in combination with the gravitational force due to the supine positioning of the patient, led to the repositioning of the subluxated IOL with the optic capture.

## CONCLUSION

Currently, there is no consensus regarding the appropriate technique to address in-the-bag IOL dislocation. Several surgical procedures have provided positive results. However, most investigators agree that it is preferable to preserve and reposition the existing IOL without surgery, avoiding the risk of potential surgical complications.

A combination of topical brimonidine and prednisolone can be an alternative treatment for an uncomplicated in-the-bag IOL dislocation, when supine positioning and intensive mydriasis have failed, before pro ceeding to more aggressive IOL manipulation and surgical repositioning of the IOL.
